# Effect of fresh pomegranate juice on the level of melatonin, insulin, and fasting serum glucose in healthy individuals and people with impaired fasting glucose

**DOI:** 10.1002/fsn3.1344

**Published:** 2019-12-17

**Authors:** Saleem A. Banihani, Reham A. Fashtaky, Seham M. Makahleh, Zeyad J. El‐Akawi, Omar F. Khabour, Nesreen A. Saadeh

**Affiliations:** ^1^ Department of Medical Laboratory Sciences Jordan University of Science and Technology Irbid Jordan; ^2^ Department of Physiology and Biochemistry Jordan University of Science and Technology Irbid Jordan; ^3^ Departement of Internal Medicine Jordan University of Science and Technology Irbid Jordan

**Keywords:** impaired fasting glucose, insulin, melatonin, pomegranate juice

## Abstract

Pomegranate juice (PGJ) is rich in unique bioactive compounds that can be used in the management of various diseases/disorders such as cancer, heart disease, Alzheimer disease, hypertension, and diabetes. Here, we aimed to investigate the effects of fresh PGJ on levels of melatonin, insulin, and fasting blood glucose in people with impaired fasting glucose (IFG). The study was a randomized clinical trial in which 28 participants (10 males, 18 females) with IFG were recruited from Irbid Central Laboratory and the Diabetes Clinic of the University Hospital at Jordan University of Science and Technology. Blood specimens from each participant were collected before (−5 min), and 1 and 3 hr after PGJ administration at 1.5 ml/kg of the body weight, and melatonin, insulin, and glucose were measured. People with IFG, but not healthy individuals, had significant antihyperglycemic response (*p* < .0001) to PGJ 3 hr after ingesting the juice. This response was not correlated with the age of participants (*p* = .4287). In addition, homeostatic model assessment of insulin resistance was significantly decreased (*p* < .0001) among people with IFG 3 hr after ingesting the juice. Moreover, 1 hr after PGJ administration, decreases in melatonin and increases in insulin were significantly observed among healthy (*p* = .0284, *p* = .0017) and IFG (*p* = .0056, *p* = .0007) individuals, respectively. In conclusion, fresh PGJ lowers melatonin, increases the level of insulin, and ameliorates insulin resistance in people with IFG.

## Graphical abstract

Fresh pomegranate juice (PGJ) was found to lower melatonin. Fresh PGJ was found to increase the level of insulin and ameliorate insulin resistance in people with impaired fasting glucose.

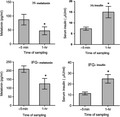

## 1 INTRODUCTION

Pomegranate (*Punica granatum *Linn) juice (PGJ) and its derived compounds can be used in the management of various diseases/disorders such as cancer (Sharma, McClees, & Afaq, 2017), heart disease (Wang et al., 2018), Alzheimer disease (Braidy et al., 2016), hypertension (Stowe, 2011), brain ischemia (West, Atzeva, & Holtzman, 2007), and diabetes (Banihani, Swedan, & Alguraan, 2013). The therapeutic effect of PGJ is attributable to the presence of several bioactive compounds that include polyphenols, flavonoids, unique sugars, and bioactive organic acids. Such compounds have antioxidant (Banihani, Shuaibu, Al‐Husein, & Makahleh, 2019; Di Stefano et al., 2018), anti‐carcinogenic (Sharma et al., 2017), anti‐atherogenic (Rosenblat, Volkova, Borochov‐Neori, Judeinstein, & Aviram, 2015), anti‐inflammatory (Cho & Song, 2018), antihypertensive (Stowe, 2011), and antihyperglycemic effects (Banihani et al., 2014).

In 2007, Katz and co‐workers have directly reported the impact of pomegranate extracts on type 2 diabetic conditions (Katz, Newman, & Lansky, 2007). A later report in 2013 done by Medjakovic and Jungbauer highlighted the potential use of pomegranate in the management of metabolic syndrome [4]. In our previous review entitled “pomegranate and type 2 diabetes,” we have described the mechanisms by which pomegranate and its derived compounds affect diabetic conditions, particularly insulin resistance (Banihani et al., 2013).

A recent study has revealed positive effects of fresh PGJ administration in enhancing betta cell function and ameliorating insulin resistance in patients with type 2 diabetes (Banihani et al., 2014). The aim of the current investigation was to study the effects of fresh PGJ on impaired fasting glucose (IFG) conditions (also known as prediabetes), given that this phase in diabetes progression is the target for many researchers in the field to prevent and manage the onset of the disease. In addition, the study measured the direct effects of fresh PGJ on the level of melatonin hormone (N‐acetyl‐5‐methoxytryptamine), as a superior controller in many physiological processes in the body, including energy metabolism (Wolden‐Hanson et al., 2000). In fact, melatonin has been shown to play a role in diabetes prevention and treatment (Rahimi, Nikfar, Larijani, & Abdollahi, 2005). Melatonin is secreted from the pineal gland and has a major role in sleep–wake timing (Tan, Xu, Zhou, & Reiter, 2018) and is confirmed as a potent antioxidant molecule (Zhang & Zhang, 2014).

## 2 MATERIALS AND METHODS

### 2.1 Subjects

This study was a randomized clinical trial in which 28 human subjects (10 adult males, and 18 adult females), with IFG, were recruited from Irbid Central Laboratory and the Endocrinology Department at King Abdullah University Hospital. The subjects were aged between 29 and 56 years; five of them were hypertensive. In addition, 28 healthy subjects as a control group (10 male and 18 female), with ages between 28 and 59 years, were also included in this study. Recruited subjects were apparently healthy and considered as having IFG if their 12‐hr fasting serum glucose was located between 6.1 and 7.0 mmol/L. All participants with IFG were selected according to the 2006 ADA criteria (American Diabetes Association, A., 2006).

Exclusion criteria were renal/hepatic disorders, pregnancy, sleep‐disorders, night shift workers, and treatment with hormonal therapy. In addition, exclusion criteria included the use of antioxidant supplements, tobacco smoking, lipid‐lowering drugs, metformin (Glucophage), melatonin medications, and glibenclamide or other oral antihyperglycemic agents.

### 2.2 Pomegranate juice

Fresh pomegranates fruits were obtained from Kofor‐Soom valley, Irbid governorate, Jordan. Fruits were stored in special cardboard boxes, not more than two weeks, for use. Pomegranate arils were separated by hand, and preparation of PGJ was performed using a squeezing machine (Philips, Japan).

### 2.3 Intervention

Blood specimens were collected in plain vacutainer tubes in the morning time (8:00–8:30 a.m.) after an overnight fasting period (mean 12 ± 1 hr). The participants from both groups (healthy and people with IFG) were given fresh PGJ (1.5 ml/kg body weight). This amount was selected randomly considering the amount that people, in the north of Jordan, consume from fresh PGJ in the small fruit juices restaurants. The allotted amount of PGJ, for each participant, was taken within 5‐min time frame. Blood specimens were obtained from participants after 1 hr and 3 hr from the zero time of PGJ administration. Then, blood specimens were centrifuged, directly after blood clotting (~15‐ to 20‐min clotting time), and each specimen was stored in several aliquots at −35°C for hormonal analysis.

### 2.4 Clinical chemistry measures

Blood glucose concentrations were determined in the Health Center Laboratory at JUST by Accent 200‐kits from PZ Cormay S.A. Diagnostics and a Chemistry Analyzer (BS‐200, Shenzhen Mindray Electronics). Insulin was measured using the Access 2 kit from Beckman Coulter. All gathered results are means of duplicate measurements. Insulin was measured using commercially chemiluminescent immunoassay kits (Access 2 Beckman Coulter). All gathered results are means of duplicate measurements.

Serum melatonin was determined using an immunoassay kit (DRG Melatonin ELISA) that used for measurement of melatonin levels in serum samples. An immunoassay‐based system was used for the quantitative determination of melatonin (supplied by Bio‐TEK instruments INC.). Before the quantitation, samples were passed through a reversed‐phase column. The collected filtrate was extracted with methanol, dried by evaporation, and dissolved in distilled H_2_O. All serum samples were assayed in duplicates, and the mean values were considered for final statistical analysis (Lahiri, Ge, Sharman, & Bondy, 2004).

### 2.5 Statistical analysis

Two‐group comparisons were performed using the Student paired *t* test, whereas ANOVA was used for multiple group comparisons. The correlation analysis was performed using Spearman's test. The GraphPad Prism software (version 6.0) was used to prepare figures and to draw statistical significance at *p*‐value < 0.05 threshold level. Glucose, insulin, and melatonin measurements were expressed as mean ± SEM.

## 3 RESULTS

**Figure 1:**
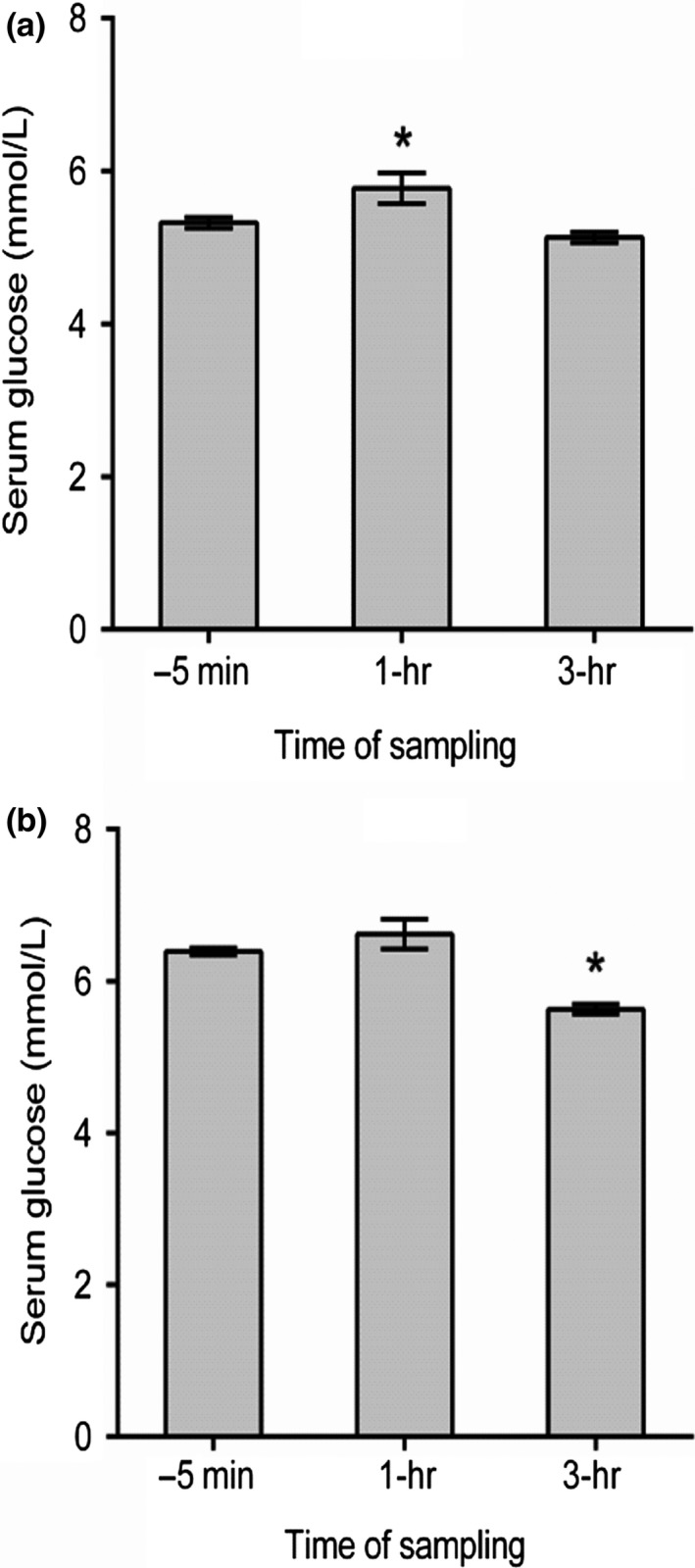
Effect of fresh PGJ on fasting serum glucose in healthy individuals (a, *n* = 28) and people with IFG (b, *n* = 28). The serum glucose was measured before (−5 min) and after PGJ dose (1.5 ml/kg, 1 and 3 hr). Measures are presented as mean ± SEM. (* *p* < .05). IFG, impaired fasting glucose; PGJ, pomegranate juice

Figure 1 demonstrates the effect of fresh PGJ on fasting serum glucose in healthy individuals (A) and people with IFG (B). The serum glucose was measured before (−5 min) and after 1 and 3 hr following the juice administration of 1.5 ml per kg of body weight. As demonstrated in the figure, serum glucose decreased significantly (*p* < .0001) after 3 hr of PGJ ingestion among IFG group, but not in healthy group.

**Figure 2:**
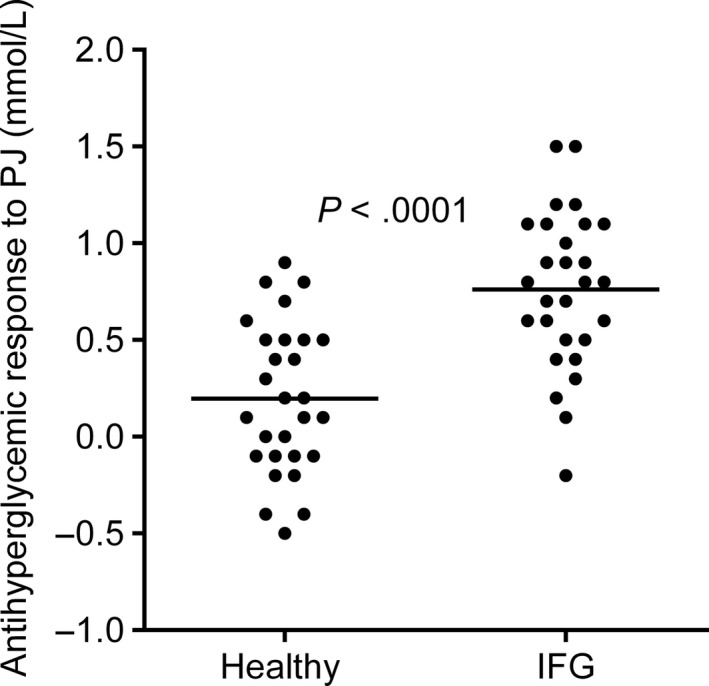
Antihyperglycemic response to fresh PGJ in healthy subjects versus people with IFG 3 hr after the juice administration. IFG, impaired fasting glucose; PGJ, pomegranate juice

Moreover, the mean antihyperglycemic response to fresh PGJ, which is the concentration of fasting serum glucose before drinking PGJ (−5 min) subtracted from glucose 3 hr after the juice ingestion, was significantly higher (*p* < .0001) in IFG group compared with healthy subjects (Figure 2). About 96.4% (27) of participants with IFG had a positive antihyperglycemic response to PGJ.

**Figure 3:**
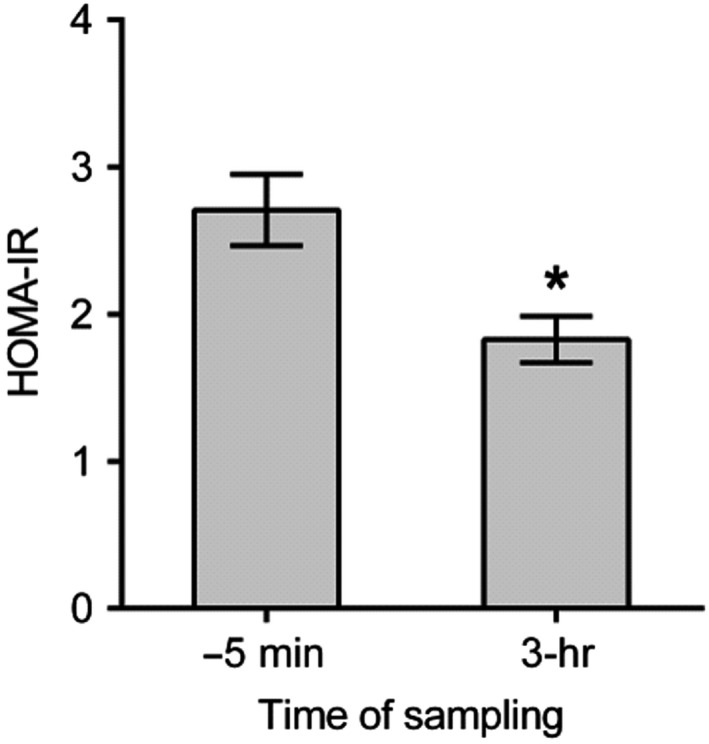
Levels of HOMA‐IR among fasted (−5 min) subjects with IFG 3 hr after PGJ dose (1.5 ml/kg). Measures are presented as mean ± SEM. (*n* = 28) (*p < .05). IFG, impaired fasting glucose; PGJ, pomegranate juice

Figure 3 illustrates the levels of homeostatic model assessment of insulin resistance (HOMA‐IR) among fasted subjects with IFG before (−5 min) and 3‐hr level after consumption of fresh PGJ of 1.5 ml per kg of body weight. The HOMA‐IR was significantly lower among subjects with IFG 3 hr after drinking fresh PGJ versus fasted individuals.

**Figure 4:**
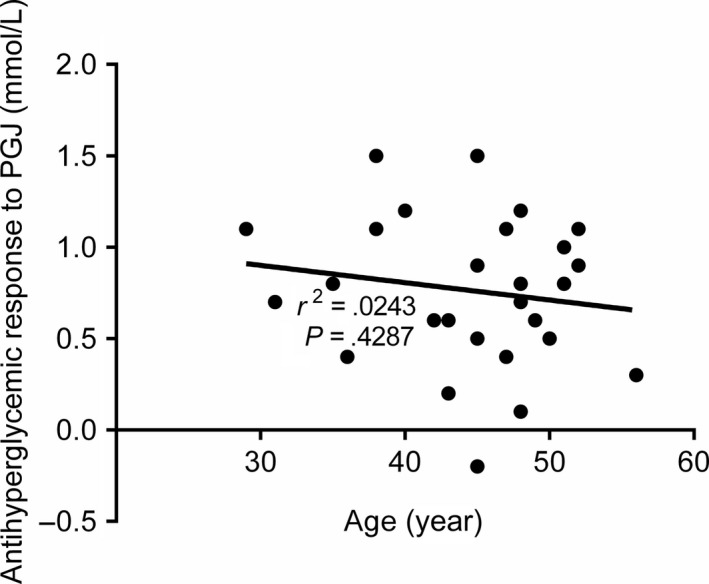
Correlation between antihyperglycemic responses to PGJ versus age in individuals with IFG. IFG, impaired fasting glucose; PGJ, pomegranate juice

Figure 4 shows the correlation between antihyperglycemic response to PGJ versus the age of individuals with IFG. No significant correlation (*p* = .4287, *r* ^2^ = .0243) was found between the antihyperglycemic response and the age of individuals with IFG.

**Figure 5:**
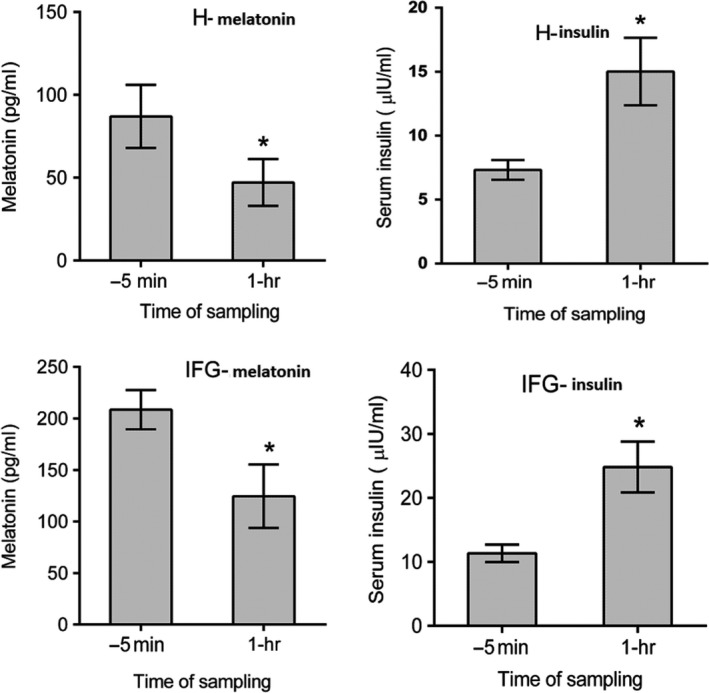
Effects of PGJ on melatonin and insulin serum levels in healthy (H‐melatonin and H‐insulin) individuals and people with IFG (IFG‐melatonin and IFG‐insulin), respectively. Serum melatonin and insulin was measured before (−5 min) and after 1 hr of PGJ consumption. Measures are presented as mean ± SEM. (*p < .05). (*n* = 18, in all experiments). IFG, impaired fasting glucose; PGJ, pomegranate juice

Figure 5 demonstrates the effects of PGJ on melatonin and insulin serum levels in healthy (H‐melatonin and H‐insulin) and subjects with IFG (IFG‐melatonin and IFG‐insulin), respectively. Serum melatonin and insulin were measured before (−5 min) and after 1 hr of PGJ consumption. As shown in the figure, melatonin was significantly decreased after 1 hr of drinking PGJ in healthy (*p* = .0284) and people with IFG (*p* = .0056).

In contrast, insulin was significantly increased in both groups (*p* = .0017, for healthy; *p* = .0007, for people with IFG) 1 hr following the juice administration.

## 4 DISCUSSION

This work is the first of its kind that demonstrates the effect of fresh PGJ and melatonin in people with IFG. In this study, fresh PGJ at 1.5 ml/kg was found to exert an antihyperglycemic response 3 hr after drinking the juice, in people with IFG, but not in healthy individuals. In addition, a decrease in insulin resistance, as assessed by HOMA‐IR, was found among people with IFG 3 hr after PGJ administration. Moreover, lower melatonin concentrations, but higher insulin concentrations, were found 1 hr following fresh PGJ administration in both groups.

In the present study, the percent of reduction in serum glucose by the effect of PGJ was approximately 11.9% in people with IFG, while this percent was approximately 3.94% in healthy individuals. These results provide some encouragement for people with prediabetes regarding fresh PGJ consumption as an alternative and safe‐dietary contribution for blood glucose homeostasis. While, individuals with diabetes must be cautious in the application (particularly for the amount) of fresh PGJ use; given that, in this study, we utilized a specific fresh PGJ dose (1.5 ml/kg of body weight). Further clinical research is still necessary to explore how fresh PGJ normalizes blood glucose level in diabetes.

The main mechanisms by which fresh PGJ affects the diabetic conditions, particularly insulin resistance ones, is by neutralizing the accumulated reactive oxygen species (ROS) such as O_2_·^−^, H_2_O_2_, and ·OH, and ultimately reducing the cellular oxidative stress (Banihani et al., 2013), an imbalance between oxidants, mainly ROS (Alzoubi et al., 2019), and antioxidants to the favor of the former (Banihani, 2018; Mahmoodi, Koohpeyma, Saki, Maleksabet, & Zare, 2019). Among the identified active antioxidant compounds in PGJ are cyanidin‐3,5‐O‐diglucoside, pelargonidin‐3,5‐O‐diglucoside, ellagitannins, punicalagin, and punicic acid (Di Stefano et al., 2019; Ding et al., 2017; Russo et al., 2018; Shabbir et al., 2017). PGJ including its active punicalagin compound was found to enhance the activity of enzymes involved in oxidants detoxification such as catalase, peroxidase, SOD, and GSH reductase (GR) (Rock et al., 2008; Rozenberg, Howell, & Aviram, 2006). Moreover, PGJ including its active compound punicic acid affects glucose homeostasis by modulating the activity of certain transcriptional factors (e.g., peroxisome proliferator‐activated receptor γ, and nuclear factor κB) (Banihani et al., 2013; Hontecillas, O'Shea, Einerhand, Diguardo, & Bassaganya‐Riera, 2009; Schubert, Neeman, & Resnick, 2002). Further, known bioactive compounds in PGJ such as punicalagin, gallic acid, ellagic acid, ursolic acid, oleanolic acid, and uallic acid have been recognized as having antidiabetic effects (Banihani et al., 2013).

Studies have shown that higher levels of resistin may lead to increased insulin resistance and reduced glucose uptake (Makino‐Wakagi et al., 2012), which ultimately leads to the accumulation of glucose in the blood, and a case of hyperglycemia (Banihani, Abu‐Alia, Khabour, & Alzoubi, 2018). PGJ, particularly ellagic acid in PGJ, was found to reduce the production of resistin hormone, a peptide‐signaling macromolecule secreted from adipose tissues (Makino‐Wakagi et al., 2012). In addition, our previous clinical study in 2014 revealed that fresh PGJ at the same dose used in this study has the ability to enhance β‐cell function in patients with type 2 diabetes (Banihani et al., 2014). The evidence above explains why fresh PGJ has an impact on fasting serum glucose conditions, rather than normal conditions; given that, such conditions are more susceptible to cellular oxidative stress and hence more sensitive to antihyperglycemic effects.

Several previous studies on pomegranate and its derived compounds are in line with our findings in this study. In animal model of diabetes, rats that fed pomegranate seed at ~450 mg/kg showed lower blood glucose level compared with control group at the end of 12 hr (Das et al., 2001). In addition, punicic acid (or trichosanic acid), a polyunsaturated organic acid present in pomegranate, at 1/100 g of the fed diet, for 30 days, was found to significantly decrease the level of fasting blood glucose in genetically obese db/db mice (Banihani et al., 2013; Hontecillas et al., 2009). Moreover, other bioactive compounds in PGJ, particularly the organic acids, ellagic, ursolic, gallic, uallic acids, and oleanolic, have been recognized as having antihyperglycemic effects (Bektas & Ozturk, 2012; Benalla, Bellahcen, & Bnouham, 2010; Fuhrman, Volkova, & Aviram, 2010; Johanningsmeier & Harris, 2011). Further, rutin, a flavonoid present in PGJ, was found to have a significant antihyperglycemic effect (Ghorbani, 2017). The mechanisms by which rutin exerts such effect may be by decreasing carbohydrates absorption from the intestine, stimulating insulin secretion, inhibiting gluconeogenesis, and increasing glucose uptake in bodily tissues (Ghorbani, 2017).

Such bioactive compounds in PGJ could be behind the observed antihyperglycemic response in people with IFG. Furthermore, compared with other fruits such as grape (Bolton, Heaton, & Burroughs, 1981; Rozenberg et al., 2006), PGJ was found to contain unique sugars that have a potent antioxidant activity (Banihani et al., 2013; Rozenberg et al., 2006). Rozenberg et al. (2006) concluded that white‐grape juice sugar fraction increases macrophage oxidative stress, while PGJ sugar fraction decreases it (Rozenberg et al., 2006). Among the bioactive compounds in PGJ are the potent antioxidant polyphenols tannins and anthocyanins (Rozenberg et al., 2006) that have been shown to enhance cellular glucose uptake in patients with type 2 diabetes (Guo & Ling, 2015). These results are in line with our findings that PGJ ameliorates insulin resistance in subjects with IFG.

In the current study, we were curious to investigate the relationships between the observed antihyperglycemic effects of fresh PGJ and the age of individuals with IFG. Actually, even though there was a decrease in the antihyperglycemic response versus age of individuals with IFG, but this decrease was not statistically significant. In our previous study on patients with type 2 diabetes, we have found a significant negative correlation between the antihyperglycemic effect of PGJ and the age of patients (Banihani et al., 2014). Such paradox may due to the use of larger sample size and larger population with higher hyperglycemic index (patients with type 2 diabetes). In fact, the antihyperglycemic effect of PGJ is more sensitive in people with higher fasting serum glucose levels (Banihani et al., 2014).

Independently, in this study, given that melatonin is involved in the glucose homeostasis and that is naturally found in pomegranate at 13–29 ng/100 g (Badria, 2002), then we asked whether PGJ consumption affects the level of serum melatonin. The results from this study suggested a decrease in the level of melatonin after 1 hr of PGJ consumption. The percent of reduction in melatonin was approximately 40.8% in people with IFG, while this percent was about 45.8% in healthy individuals. In contrast, insulin levels found to increase after 1 hr of PGJ administration. The percent of insulin 1 hr after PGJ administration was approximately doubled in both tested groups. Studies have shown that the circadian rhythm of melatonin affects the mode of insulin secretion from the pancreas (Peschke et al., 2006; Elmar Peschke et al., 2007). Specifically, melatonin was found to have a suppressive effect on β‐cell activity (Rasmussen, Boldt, Wilkinson, Yellon, & Matsumoto, 1999; Rasmussen, Mitton, Larsen, & Yellon, 2001), such effect agrees with a decrease in glucose tolerance (Cagnacci et al., 2001; Dhar, Dayal, Ramesh, & Arora, 1983). Vice versa, increased insulin concentration was found to inhibit the secretion of melatonin from the pineal gland (Champney, Brainard, Richardson, & Reiter, 1983; Champney, Steger, Christie, & Reiter, 1985). Therefore, a “functional antagonism” between melatonin and insulin might exist (Boden, Ruiz, Urbain, & Chen, 1996; Peschke et al., 2011). The present findings are in line with the above evidence. Thus, the observed improvements in glycemic control among people with IFG could be due to, at least in part, the effect of PJG on circulatory melatonin and insulin levels.

The findings of the current study showed a decrease in HOMA‐IR and hence in insulin resistance, among people with IFG 3 hr after PGJ administration. In general, the calculation of HOMA‐IR level 3 hr after consumption of the juice might not be appropriate in nonfasting state; however, participants were not allowed to consume any food during the experiment and the juice was assumed as a treatment given during the fasting period. Besides, insulin resistance was assessed 3 hr following the juice ingestion, a state where serum glucose level has been found to be significantly decreased even in patients with type 2 diabetes (Banihani et al., 2014). In fact, this approach was used in previous studies (Banihani et al., 2014; Papandreou et al., 2019) and we use it in the current study to gather additional evidence to support the effectiveness of FPG on the management of type 2 diabetes.

Among the limitations of the present study is that we did not include PJG‐free groups. A circadian rhythm in circulatory melatonin level has been reported (Kennaway & Voultsios, 1998). Therefore, the possibility that the observed decrease in melatonin levels after consumption of PJG is due to circadian clock rather than the action of the juice cannot be excluded. Therefore, more investigation is required to explore such possibility.

In conclusion, fresh PGJ (1.5 ml/kg) was found to have an antihyperglycemic response, after 3 hr of ingesting the juice, in people with IFG. The response was not correlated with the age of people with IFG. Additionally, a decrease in HOMA‐IR, and hence in insulin resistance, was found among people with IFG 3 hr after PGJ administration. Besides, lower melatonin levels, but higher insulin levels, were observed 1 hr following PGJ administration in both healthy and people with IFG.

## CONFLICTS OF INTEREST

The authors declare no conflict of interest.

## ETHICAL STATEMENT

A written informed consent was given to each participant before enrolled in the study. This clinical trial was approved by Institutional Review Board of KAUH/Jordan University of Science and Technology. In addition, it was registered in ClinicalTrials.gov (Identifier: NCT03902288).
